# Needle to needle robot‐assisted manufacture of cell therapy products

**DOI:** 10.1002/btm2.10387

**Published:** 2022-08-06

**Authors:** Jelena Ochs, Mariana P. Hanga, Georgina Shaw, Niamh Duffy, Michael Kulik, Nokilaj Tissin, Daniel Reibert, Ferdinand Biermann, Panagiota Moutsatsou, Shibani Ratnayake, Alvin Nienow, Niels Koenig, Robert Schmitt, Qasim Rafiq, Christopher J. Hewitt, Frank Barry, J. Mary Murphy

**Affiliations:** ^1^ Fraunhofer Institute for Production Technology (IPT) Aachen Germany; ^2^ School of Biosciences, Life and Health Sciences College Aston University Birmingham UK; ^3^ Chemical Engineering University College London London UK; ^4^ Regenerative Medicine Institute, Biomedical Sciences Building National University of Ireland Galway Galway Ireland; ^5^ Chemical Engineering University of Birmingham Birmingham UK; ^6^ Faculty of Mechanical Engineering RWTH Aachen University Aachen Germany; ^7^ Biochemical Engineering, Advanced Centre for Biochemical Engineering University College London London UK

**Keywords:** automation: bioreactors, cell therapy, human stem cells, manufacturing

## Abstract

Advanced therapeutic medicinal products (ATMPs) have emerged as novel therapies for untreatable diseases, generating the need for large volumes of high‐quality, clinically‐compliant GMP cells to replace costly, high‐risk and limited scale manual expansion processes. We present the design of a fully automated, robot‐assisted platform incorporating the use of multiliter stirred tank bioreactors for scalable production of adherent human stem cells. The design addresses a needle‐to‐needle closed process incorporating automated bone marrow collection, cell isolation, expansion, and collection into cryovials for patient delivery. AUTOSTEM, a modular, adaptable, fully closed system ensures no direct operator interaction with biological material; all commands are performed through a graphic interface. Seeding of source material, process monitoring, feeding, sampling, harvesting and cryopreservation are automated within the closed platform, comprising two clean room levels enabling both open and closed processes. A bioprocess based on human MSCs expanded on microcarriers was used for proof of concept. Utilizing equivalent culture parameters, the AUTOSTEM robot‐assisted platform successfully performed cell expansion at the liter scale, generating results comparable to manual production, while maintaining cell quality postprocessing.

## 1 INTRODUCTION

Advanced therapy medicinal products (ATMPs) include cell and gene therapies with demonstrated clinical efficacy for previously untreatable conditions. Cell therapy involves delivery of living autologous or allogeneic cells to promote tissue repair, modulate inflammation or correct an autoimmune environment. However, ex vivo, large‐scale cell manufacturing requires production platforms enabling cost‐effective expansion to generate clinically relevant numbers.^1^ This prerequisite presents significant challenges that are constrained by current manual, open, small‐scale production methodologies/systems. Another impediment to broad clinical use includes the numerous early‐stage ATMP candidates which require human operators to undertake critical functions and are driven by academic sites, clinical centers and SMEs which lack resources and expertise to automate and scale manufacturing processes.^2^

The use of cell‐based manufacturing systems for human medicines has led to successful outcomes in recent decades. Recombinant protein drugs (cytokines/monoclonal antibodies) are produced using various expression systems inserted into immortalized CHO cells, capable of unlimited expansion at scale. A new challenge arises when the therapeutic product is the cell itself rather than its protein product. Cell therapies use expanded nonimmortalized, primary cells with complex biological characteristics, whether autologous or allogeneic. Despite these obstacles, the field has made enormous strides in recent years with many outstanding examples, for example, the use of mesenchymal stromal cells (MSCs) for immune disorders or degenerative conditions such as Crohn's disease and osteoarthritis. More recently, MSCs were tested in patients with acute respiratory distress syndrome associated with SARs‐CoV‐2 infection.^3^

The emergence of efficient production systems for clinical grade MSCs using scalable bioreactors has been slow. Current production systems mainly involve laboratory‐scale manual processing using flasks or cell factories and the dispersed nature of such manufacturing is associated with exceptionally poor process standardization.^4^ Critical process parameters including cell source, media composition, seeding density, passage number, and final dose formulation are highly variable. Additionally, process checkpoints and quality control are not uniformly applied and industry standards for release criteria and potency testing have not yet emerged. Current methods generally require full grade A containment levels for qualification, sterility control and maintenance contributing to the very high costs associated with approval and market entry.

There is an urgent need for highly efficient automated manufacturing platforms for the production of ATMPs for patient benefit. This recognition is driven by the pursuit to minimize process and manufacturing variation, improve product quality, minimize risk, reduce error, improve site‐to‐site comparability and ensure compliance with global regulatory standards.^1^, ^5^, ^6^ Early automated systems such as the SelecT/CompacT SelecT™ systems (Sartorius Stedim) were designed to mimic human operator actions when performing routine cell culture processes. These platforms resulted in higher process consistency allowing for the implementation of process improvement techniques such as six‐sigma,^7^ but the use of monolayer T‐flask culture led to limited scalability.^8^, ^9^

The current need for significant manual intervention in many ATMP production processes can result in significant quality and cost implications.^1^ For scale‐out approaches, numerous practical challenges are associated with manipulation, segregation, and production of multiple batches in an aseptic manner. These processes require small manufacturing units where line segregation is a priority to avoid cross‐contamination, product–patient mismatch and disease transmission.^10^ Automated production platforms such as AUTOSTEM are therefore a high priority for ATMP industrialization to improve consistency of manufacture, address issues of site‐to‐site comparability, enhance process capability and improve overall process economics.^6^, ^9^ Moreover, such platforms can be rapidly deployed to ramp‐up production of ATMPs or address limitations in manufacturing capacity at times of urgent need, as demonstrated by the demand for human (h)MSCs to treat complications of COVID‐19 with associated acute respiratory distress syndrome.^11^

With each ATMP candidate likely requiring a unique manufacturing process, there are advantages in having automated platforms with the capability to integrate and interlink modular processing components to support different processes and integrate new technologies.^12^, ^13^, ^14^ These systems can facilitate centralized and decentralized manufacture and fundamentally represent cross‐cutting platform technologies.^14^ In the platform developed, human engagement is limited to transfer of bone marrow to the production center and interactions with the process control software. The process can be monitored and controlled by sensors that facilitate real‐time online monitoring, thereby ensuring the manufacturing platform can respond to potential adverse events.

Here we describe the development and validation of this fully automated, GMP‐amenable manufacturing platform and demonstrate its functionality for the production of clinically relevant hMSCs.

## 2 RESULTS

### 2.1 AUTOSTEM platform overview

The robot‐assisted AUTOSTEM cell factory was designed to encompass the entire cell production process in a fully automated system without any direct interaction between the biological product and operators. The platform was configured as a test bed for large‐scale production of bone marrow‐derived hMSCs, given the challenges associated with their manufacture at scale.

The robotically controlled sequence embraces all cell processing steps including (1) bone marrow harvest, (2) cell isolation and expansion in bioreactors at the liter scale, (3) cell harvesting, and (4) cryopreservation of filled doses.

Equipment includes:

1. Two 3 L single‐use bioreactors for cell isolation, cell expansion on microcarriers and cell detachment from them connected to cooled or ambient reservoirs and an array of squeeze‐valve pumps for liquid transportation.
2. A cell counter for automated cell sampling and analysis.
3. An automated centrifuge, decapper and pipetting device for filling and formulation of the cell suspension.
4. A −80°C freezer for cryopreservation.

**FIGURE 1:**
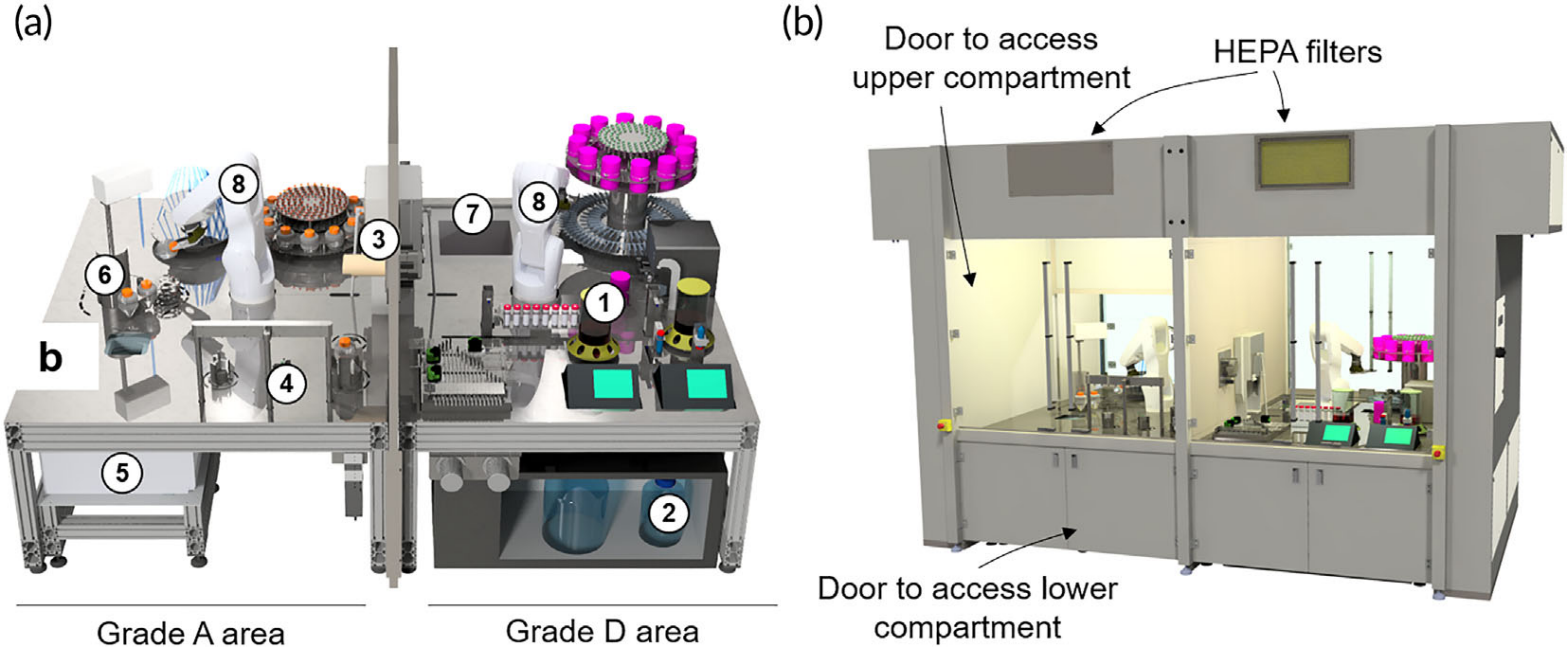
Schematic diagrams of the AUTOSTEM platform and its components: (a) internal view of the platform in Grade A and Grade D areas with the individual components. 1: Bioreactors with control units, 2: cooled medium storage, 3: material transfer, 4: decapper, 5: centrifuge below working area, 6: automated pipette, 7: −80°C freezer, 8: robot arms. (b) external view of the platform showing the hood and access points, as well as the elements necessary for achieving and maintaining cleanliness of the inside environment (e.g., HEPA filters).

The platform consists of two chambers with different clean room containment levels (A, D) that take into consideration safety requirements in cell manufacturing for therapeutic use (Figure 1a). The chamber providing a grade A environment is operated as an isolator, ultimately enabling placement of the system in a clean controlled room for compliant therapeutic production.

**FIGURE 2:**
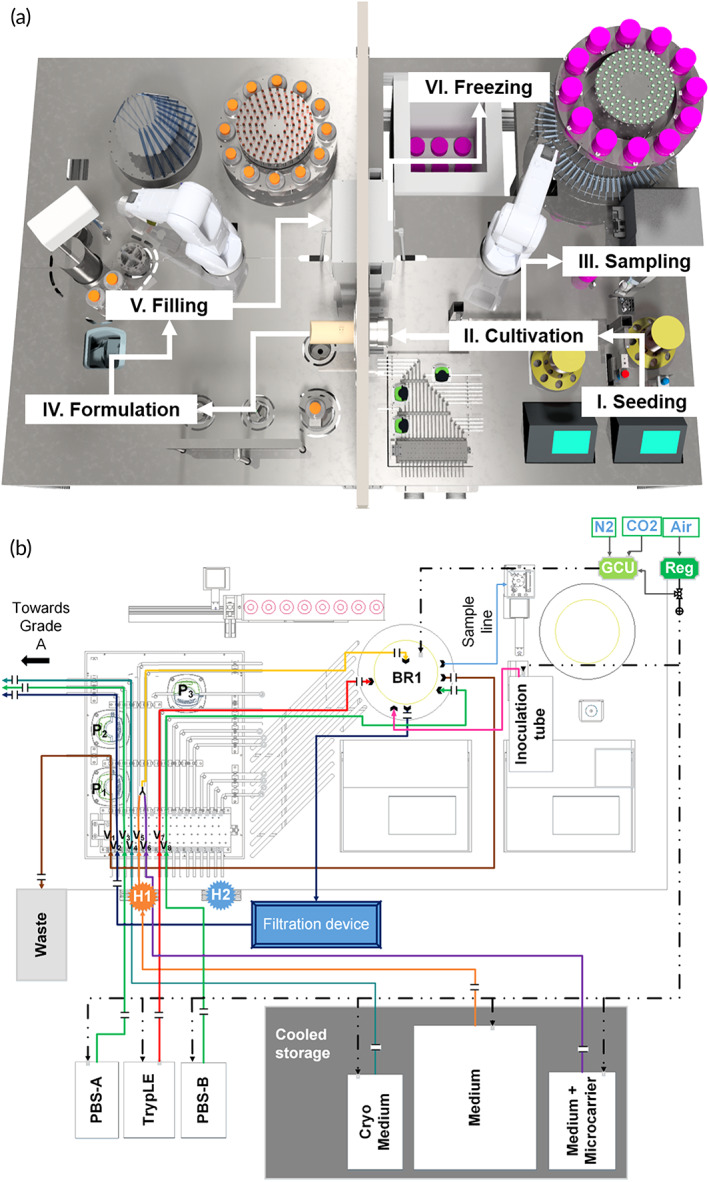
Complete manufacturing process mapped out on the AUTOSTEM system and liquid flow on the platform. (a) Illustration of the production process steps along the different stations on the facility, including I: Seeding of the cells into the bioreactor; II/III: Cultivation, Sampling and Harvesting; IV and V: Formulation and Filling to VI. Freezing of the final cell product in the −80°C freezer. (b) Illustration of the tubing organization between bioreactor, media reservoir, waste and grade A area. For better usability, each line has been assigned a different color. Solid lines: liquid; dashed lines: gas; H1: Heater 1; H2: Heater 2; BR1: Bioreactor 1; GCU: gas control unit, Reg: gas regulator; P1‐3: Peristaltic pump 1–3, V1‐8: squeeze valve 1–8.

Upstream processing, including isolation of seeding cells from tissue and cell expansion in bioreactors as well microcarrier separation is contained in enclosed vessels (single use tubes/bioreactors) and performed at the lower grade D cleanroom level. However, formulation and filling of harvested cells into cryovials, requiring open processing steps, is implemented in the grade A area to ensure product sterility. Both areas contain an industrial six‐axis robot with custom‐designed gripper tools for handling and transportation of material and disposables (Figure S1.4, S1.5, S1.8, S1.9). The system is equipped to perform cell cultivation from seeding to harvesting in the bioreactor, as well as automated sampling and freezing of cryovials in the grade D area; the grade A area is equipped for the formulation and filling of cell suspensions (Figure 2a and Video S1).

### 2.2 Automated bone marrow collection device

**FIGURE 3:**
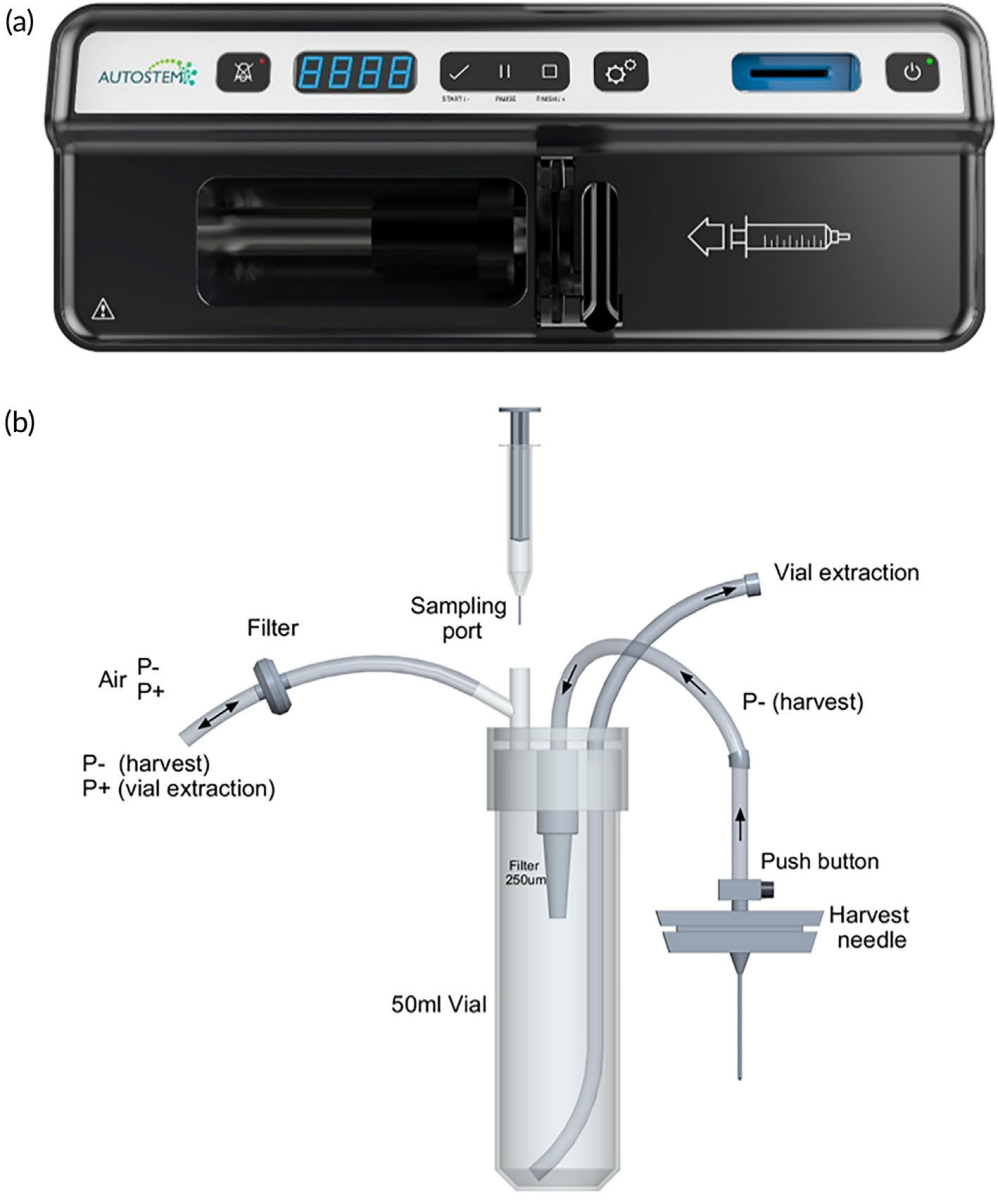
Automated bone marrow collection device. (a) External device to operate the automated collection. (b) Disposable tube set for marrow harvest, including a harvest needle, a sampling port and a port for connection to the AUTOSTEM platform and vial extraction.

A novel system for extracting bone marrow from patients with a key design objective to maintain sterility of the marrow and minimize manual handling of the sample was developed (Cro‐spon Ltd.) (Figure 3a). A pump facilitated optimal control of vacuum pressure and a disposable tube set connected to a standard biopsy needle for marrow harvest delivered the sample to the platform through a direct connection to the bioreactor for fast inoculation.

### 2.3 Sterility

Sterility and containment in both processing areas is ensured through a series of aseptic control points. Both areas are equipped with integrated HEPA filters and laminar air flow (Figure 1b). Grade A is operated as a positive pressure environment to prevent ingress of external air and particles. In line with isolator principles, this area is sterilized between production runs by vaporized hydrogen peroxide (VHP) using dedicated inlet and outlet ports. Additionally, the doors of the system can be opened to access the surfaces between production campaigns to enable cleaning of surfaces and equipment with additional manual wipe‐down if deemed necessary. Validation will be performed after cleaning between production campaigns to ensure GMP compliance.

Required single‐use materials (e.g., centrifuge tubes/cryovials) for operation are loaded manually into the system prior to operation. Each area has its own robot‐accessible material warehouse (Figure S1.4). Upon initial material stocking, the warehouse for the grade A isolator is separated by a retractable partition wall (not shown). This ensures that only the storage area is opened for stocking of bagged disposables, leaving the main area uncompromised. After gassing both chambers, the user unpacks the disposable bags from the outside through wall‐mounted gloves and places them into the robot‐accessible material warehouse. Subsequently, the retractable partition wall between main and material chamber is opened for robot access.

### 2.4 Grade D area

**FIGURE 4:**
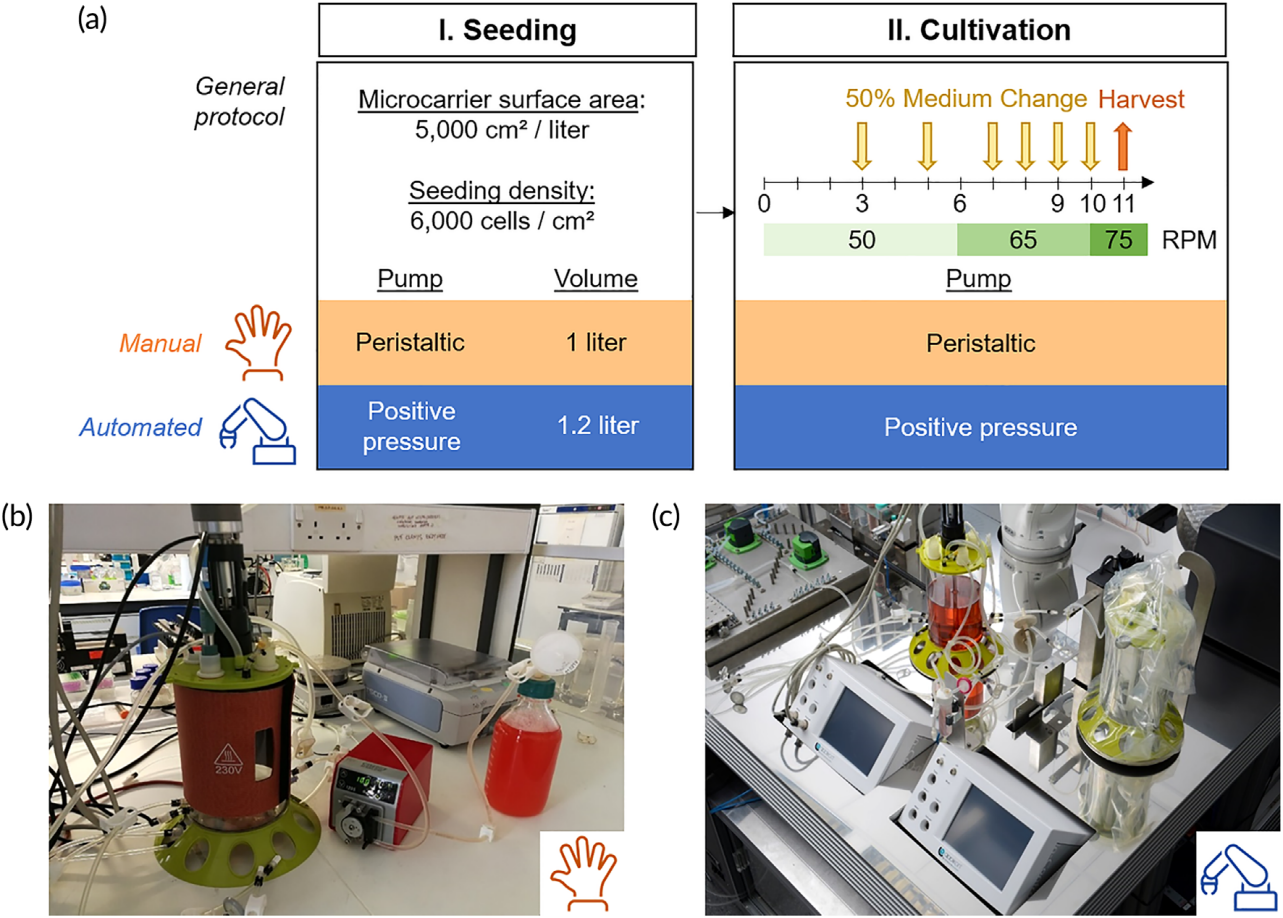
Comparison of manual and automated process protocols. (a) Sequence diagram of the manual and automated processes. The general protocol for the process is described with a comparison of the manual and automated processes. (b) Photographs of the manual bioreactor set up using filtered bottles, peristaltic pump and sterile welder. (c) Automated bioreactor set up within the AUTOSTEM platform, showing the bioreactors connected to the control units and a pumping station using both positive pressure and peristaltic pumps.

The grade D clean room is equipped with two single‐use stirred tank bioreactors (Mobius 3L; Merck Millipore) situated in close proximity to their controllers (Figure 4c). These allow parallel bioprocesses to be run concurrently or in direct sequence; for example, parallel isolation/expansion of hMSC from two different donors (Figure S1.1). Accurate metering of liquids is achieved by placing the bioreactors on a weigh scale; CO_2_/O_2_ are delivered to the bioreactors using tubing. Communication between the bioreactor controllers and the platform control level software is established through an OPC Data Access server that allows programmed commands to be entered and measurements read, stored and evaluated in real time.

Media and liquid reagents are stored below the platform at 3–7°C and transported to and from the bioreactor via sterile tube sets guided through an array of pumps and squeeze valves (Figure 2b). Positive pressure is applied to these reservoirs to facilitate liquid transport. Delivery is controlled by opening and closing tube line squeeze valves that respond to weight data from the bioreactor scales, facilitating feedback control of feed volume and rate. Three peristaltic pumps are available for liquid removal from bioreactors. Liquids from cooled reservoirs are passed through heating blocks (Figure S1.3) for temperature equilibration before entering the bioreactors, thus avoiding the introduction of temperature gradients.

#### 2.4.1 Sampling

Regular sampling during expansion, required for monitoring cell growth and nutrient/metabolite levels, is performed via a tube attached to a small peristaltic pump. The automated sampling system consists of two devices: one discards residual volume from the tube, draws a sample volume and dispenses it through a needle into a septum capped vial; the second draws reagents from an array of septum‐capped 50 ml tubes through a needle connected to a piston pump. The automated sampling system is capable of aspirating, dispensing and mixing fluids. To avoid bubble formation, the sample vial is robotically tilted (45°) to ensure the needle touches the vial wall upon dispensing.

To facilitate cell counting, robotic steps include (1) removal of the sample vial cap, (2) sample acquisition from the bioreactor and addition to the vial, (3) cap insertion of a Nucleocounter cassette (Via‐1), (4) sample collection enabled by pushing the cassette plunger, and (5) insertion of the cassette into the automated cell counter (Nucleocounter NC3000; Chemometec). A custom decapper allows robot‐assisted unscrewing and discarding of the lid. The robot places the cassette in the uncapped vial and a metal plunger pushes the cassette plunger down to aspirate cell suspension (Figure S1.7) before inserting it into the Nucleocounter.

#### 2.4.2 Cell harvest in the bioreactors

**FIGURE 5:**
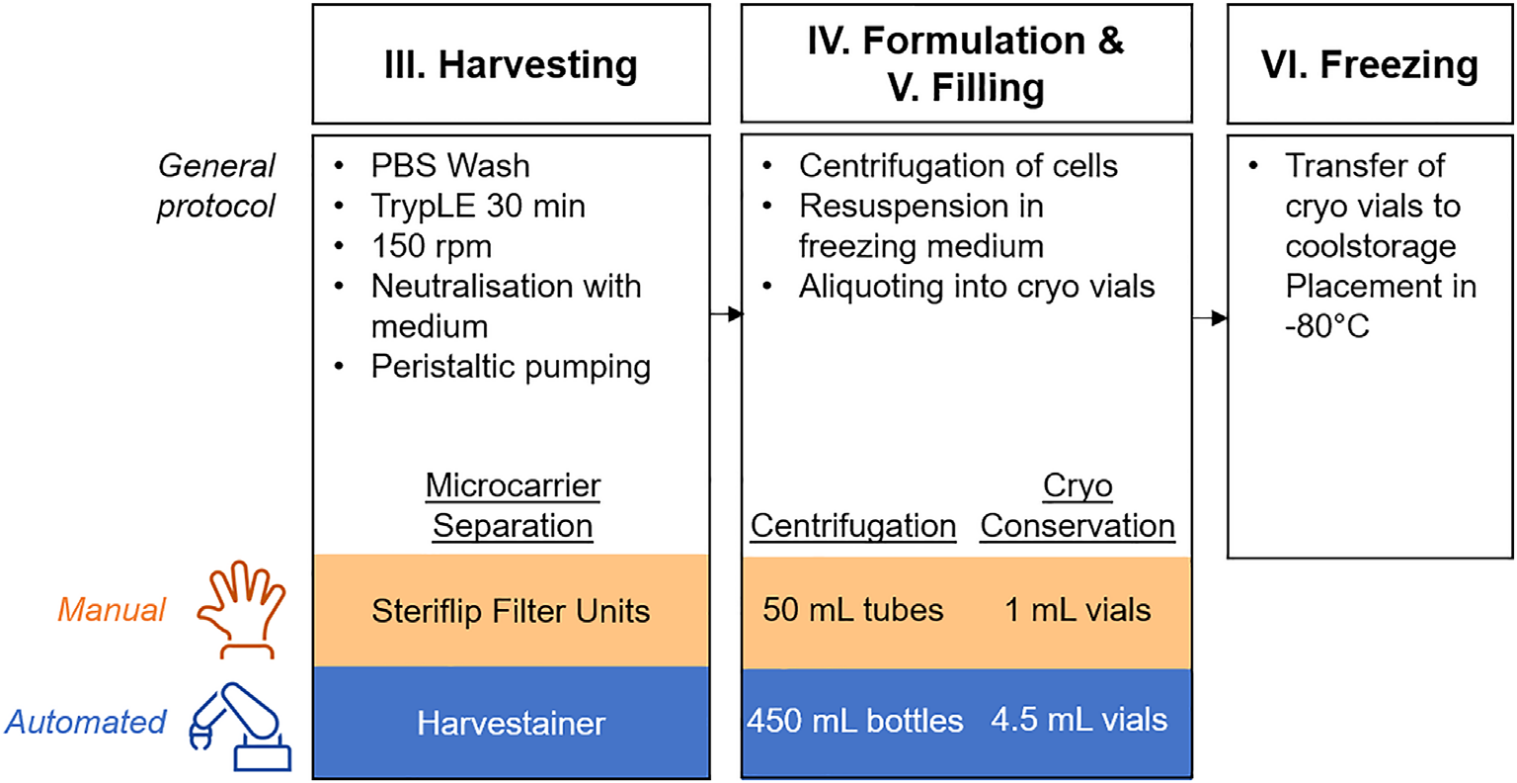
Sequence diagrams of the harvesting process. General protocol for the process with differences between the manual and automated processes highlighted.

At the end of the culture process in both the manual and robotic cultures, cells are harvested inside the bioreactor by incubation with a proteolytic enzyme plus a short period of higher intensity agitation to release the cells from the microcarriers resulting in a suspension of cells and microcarriers (Figure 5).^8^ More details are provided in Section 4.2.4. This suspension is filtered through a single‐use filter devices prior to transfer to the grade A area for downstream processing open to the environment. The filter used was different in the manual and robotic cultures (Figure 5).

### 2.5 Grade A area

#### 2.5.1 Liquid transfer

To mediate transfer of the harvested cells produced in the grade D area bioreactor, a novel device was developed for transfer to grade A conditions without compromising clean room levels. A custom‐designed adapter for rapid fluid transportation is loaded into the grade A chamber prior to sterilization at the initiation of a bioprocess run, with the robotic arm inserting the connector into a custom‐designed port in the wall between the two areas. This placement enables access by operators through the grade D area to place single‐use sterile connectors and tubing for transferring the cell suspension, while maintaining grade A area integrity.

#### 2.5.2 Capping and uncapping

Capping and uncapping a variety of containers (cryovials, centrifugation bottles, sampling vials, Cool Cell containers) is required for various bioprocessing steps with four different decappers designed and incorporated. All consist of three brackets opening and closing pneumatically to hold and grip the disposable containers. For disposables with screw cap lids (vials and bottles), the brackets are turned by a motor to facilitate uncapping, that is, the robots hold the cap and the bottle/vial is rotated. In addition, the decappers have a spring suspension allowing disposables to move up/down when turned (Figure S1.5).

The cell suspension, pumped through the liquid transport device into the grade A area, is filled into 500 ml centrifugation bottles, which sit on a holder mounted on a weigh scale for precisely measuring and controlling the dispensed volume. Once filled and recapped, the robot transfers them to the automated centrifuge below the platform.

#### 2.5.3 Formulation and filling

After centrifugation, the robot pours the supernatant into a funnel connected to a vacuum pump and a reservoir located below the platform. Through the same fluid transport connector as above, the washing buffer or cryopreservation medium can be filled into centrifugation bottles. An automated pipetting device was incorporated in the grade A area to facilitate cell resuspension, sampling and the filling of cryovials. This device utilizes standard sterile, single‐use serological pipettes and rotates around its own axis, moving up and down to aspirate or dispense liquids. Liquids can be pipetted to and from ambient or actively cooled centrifugation bottles, into 5 ml cryovials or 1 ml sampling vials. One pipetting step enables the device fill up to five 5 ml vials (Figure S1.6). In total, up to 450 ml of formulated suspension can be filled per bioreactor run.

#### 2.5.4 Transfer of cryovials

A hatch designed to transfer up to 10 cryovials and two sample vials from grade A to D (Figure S1.8) contains two doors which can be opened pneumatically, but not simultaneously, to allow access from both sides. The vial holder inside is actively cooled to aid cryopreservation. The grade A and D robots can access the hatch to enable placement or removal of the vials. After filling, the robot in the grade A area loads the hatch with up to 10 cryovials from one side. The hatch is then closed with the cryovials transferred to the freezer for cryopreservation by the grade D robot on the other side. This process is repeated multiple times until batch cryopreservation is complete.

#### 2.5.5 Cryopreservation

After the cryovials have been placed in the hatch from the grade A side, the grade D area robot transfers the vials into controlled‐rate freezing containers sitting in a retractable holder (Video S1). The robot uncaps and caps the freezing container (Cool Cell) after loading the filled cryovials, and subsequently places it into an integrated −80°C freezer that opens and closes automatically (Figure S1.9). The freezer has space for 9 freezing containers which can hold up to 10 cryovials each, thus providing a capacity for freezing 90 vials. The frozen cryovials can be manually removed from the platform for long term storage at lower temperatures.

### 2.6 Control software

The AUTOSTEM platform is operated through control level software, allowing monitoring and control of all devices via a graphical user interface (GUI, Figure S2a). The custom‐built devices (e.g., decapper, pump station) are controlled by programmable logic controllers, while commercial equipment comes with proprietary controllers. In both cases, all devices are integrated via specifically programmed software agents managing the communication between the control and hardware levels. This service‐oriented approach has been successfully applied to other lab automation systems and allows flexible control over the system independent from available interfaces.^15^ Each device offers a list of primary functions on the control level software that can be executed manually via the GUI of the control level (Figure S2b) or arranged in a recipe builder (Figure S3) for more complex sequences, including complete processes. The user can also incorporate decision trees that execute different process branches based on measurement data or user input to automate adaptive processes.

### 2.7 AUTOSTEM system validation

Validation of the AUTOSTEM system was performed through replicate experimental expansion runs from hMSC inoculation to cell harvest, both following the same bioprocess (Figure 4a) and performed contemporaneously in the manual and automated systems. An experimental run was deemed successful if sterile cell expansion was achieved on microcarriers with successful harvesting and cell quality retained postexpansion.

#### 2.7.1 Manual versus automated expansion

**FIGURE 6:**
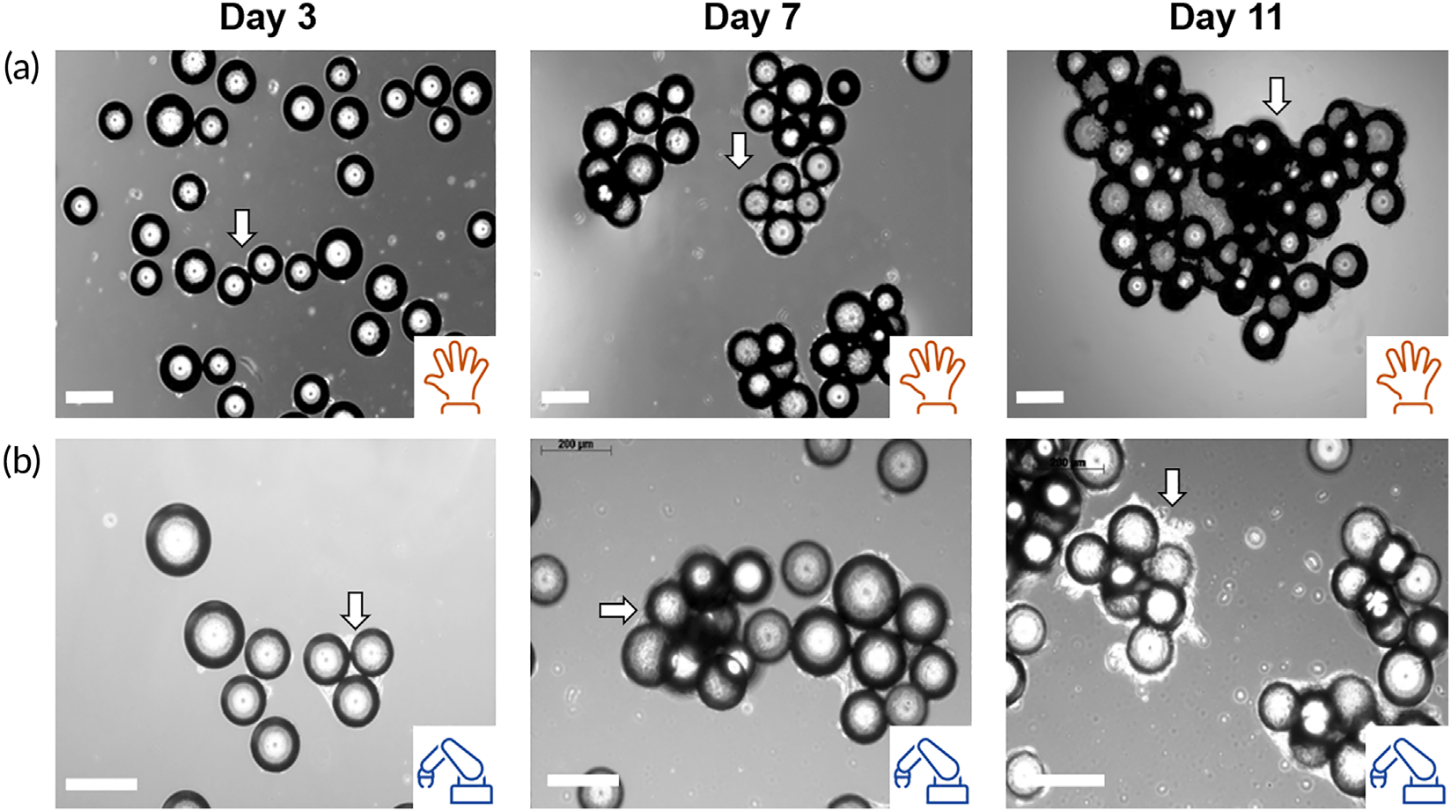
Phase contrast images of hMSCs on plastic microcarriers in the stirred tank bioreactor. Phase contrast images of hMSC cultured on Plastic microcarriers in the Mobius 3L vessel at different time points (days 3, 7 and 11) in the (a) manual and (b) automated culture processes. White arrows point to cell‐microcarrier aggregates. Scale bars, 200 μm.

**FIGURE 7:**
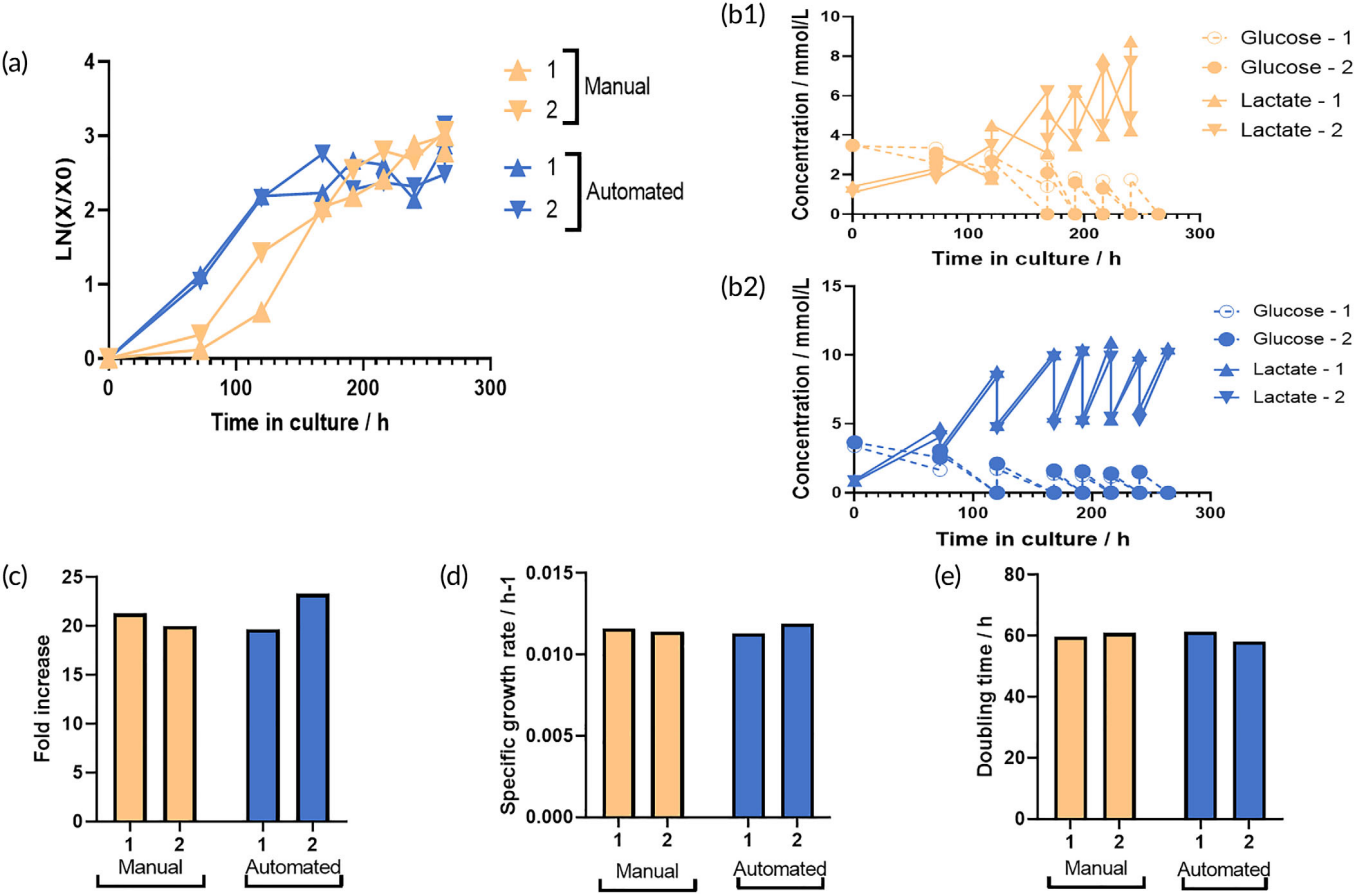
Comparison between hMSC expansion on microcarriers in the manual and automated systems. (a) hMSC growth in manual and automated systems. (b1), (b2), Glucose (G) and lactate (L) concentrations over time in the manual and automated systems, respectively. (c) Fold increase. (d) Specific growth rate (h‐1). (e) Doubling time (h). 1 and 2 represent two independent runs.

Phase contrast images acquired at different time points in culture are shown in Figure 6a for the manual and for the automated system (Figure 6b). In both systems, the cells successfully attached to the microcarriers with cell‐microcarrier bridges, indicated by the white arrows and associated with cell proliferation observed.^13^, ^16^ In the automated platform, the formation of cell‐microcarrier bridges was evident as early as day three in culture (Figure 6b). However, in the manual system at the same time point, there was no evidence of bridging (Figure 6a). Associated growth curves also show delayed growth in the manual system with a lag phase in growth observed and the growth rate slower when compared to that of the automated system up to day 3 (Figure 7a). The growth rate in the automated system continued to outperform the manual system until day 7. This growth profile data is also reflected in the trends seen for glucose and latate measurements from media sampled from the bioreactors during the culture process (Figure 7b1,b2), where the glucose levels recorded for the automated system were depleted by day 5. However for the manual system complete depletion of glucose was not evident until day 7. High lactate concentrations, approximately 10 mmol/L, were also detected in medium sampled from the automated system by day 5. This was almost double the lactate concentration found in the manual system at the same time. The trends for cell growth and the glucose and lactate profiles were consistent for both experimental runs.

Initial increased growth in the automated platform might be due to decreased holding times and lack of gradients (temperature and concentration) with AUTOSTEM designed to overcome such limitations and challenges, typically encountered during manual culture.^9^, ^12^, ^17^, ^18^ Temperature gradients were unavoidable as all manual liquid handling was carried out by peristaltic pumps at a relatively low flow rate (~50 ml/min), chosen to maintain tubing integrity. Typically, 500 ml manual medium exchanges required ~20 min. This time lag also resulted in temperature gradients within the manual bioreactor, an issue not seen in the AUTOSTEM platform as liquids are passed through a heating coil.

As cultures progressed, a growth plateau was evident beyond day 7 (Figure 7a), perhaps attributable to the increased levels of cell‐microcarrier aggregation observed. At this time, most of the available surface area provided by the microcarriers was utilized by cells leading to contact inhibition and cell growth arrest.^19^ Above a certain size, aggregation is undesirable as cells can be exposed to nutrient and oxygen concentration gradients leading to a heterogeneous cell population and even cell death. When the cells are the product, this heterogeneity can lead to concerns on the safety of the therapy.^20^

Decreased cell growth can result from nutrient depletion or metabolite inhibition.^21^, ^22^ As early as day 5 (in the AUTOSTEM system) (Figure 7b2) and day 7 (in the manual system) (Figure 7b1), glucose concentration dropped to below 1 mmol/L. The 50% medium exchange, performed daily was not able to increase the glucose concentration as a result of the high cell numbers obtained beyond day 7. This problem can be addressed through increasing the working volume to allow for a higher % of medium exchange and/or frequency of medium exchange, increasing glucose levels in the medium or incorporating an automated, sensor‐guided continuous feeding system in the platform. Overall, when comparing the automated to the manual set‐up at the end of the culture, there was no difference in fold expansion levels (Figure 7c), specific growth rates (Figure 7d), or doubling times (Figure 7e). A fold increase of approximately 20 was achieved, while the doubling time was ~60 h and specific growth rate 0.011 h^−1^.

However, the nature of the comparison performed here required that the manual and automated processes be synchronized with respect to the culture methodology as well as the timing of media supplementation or carrier beads. In the automated platform, cells showed early proliferation with a plateau in cell numbers observed from day 8 onwards associated with contact inhibition. Delayed proliferation was evident with minimal growth from day 1 to day 3 in the manual cultures and expansion evident to day 11, the point selected for cell harvest. This data highlights the potential for automated control of the bioreactor milieu to enable increased cell yields in a shorter timeframe leading to a more defined cell and decreased costs ultimately. However, the data acquired also illustrated the need to define appropriate cues for cell harvest such as increased glucose consumption and/or lactate levels, as well as clumping of the nanoparticles through cell‐to‐cell contacts.

### 2.8 hMSC quality assessment postexpansion

**FIGURE 8:**
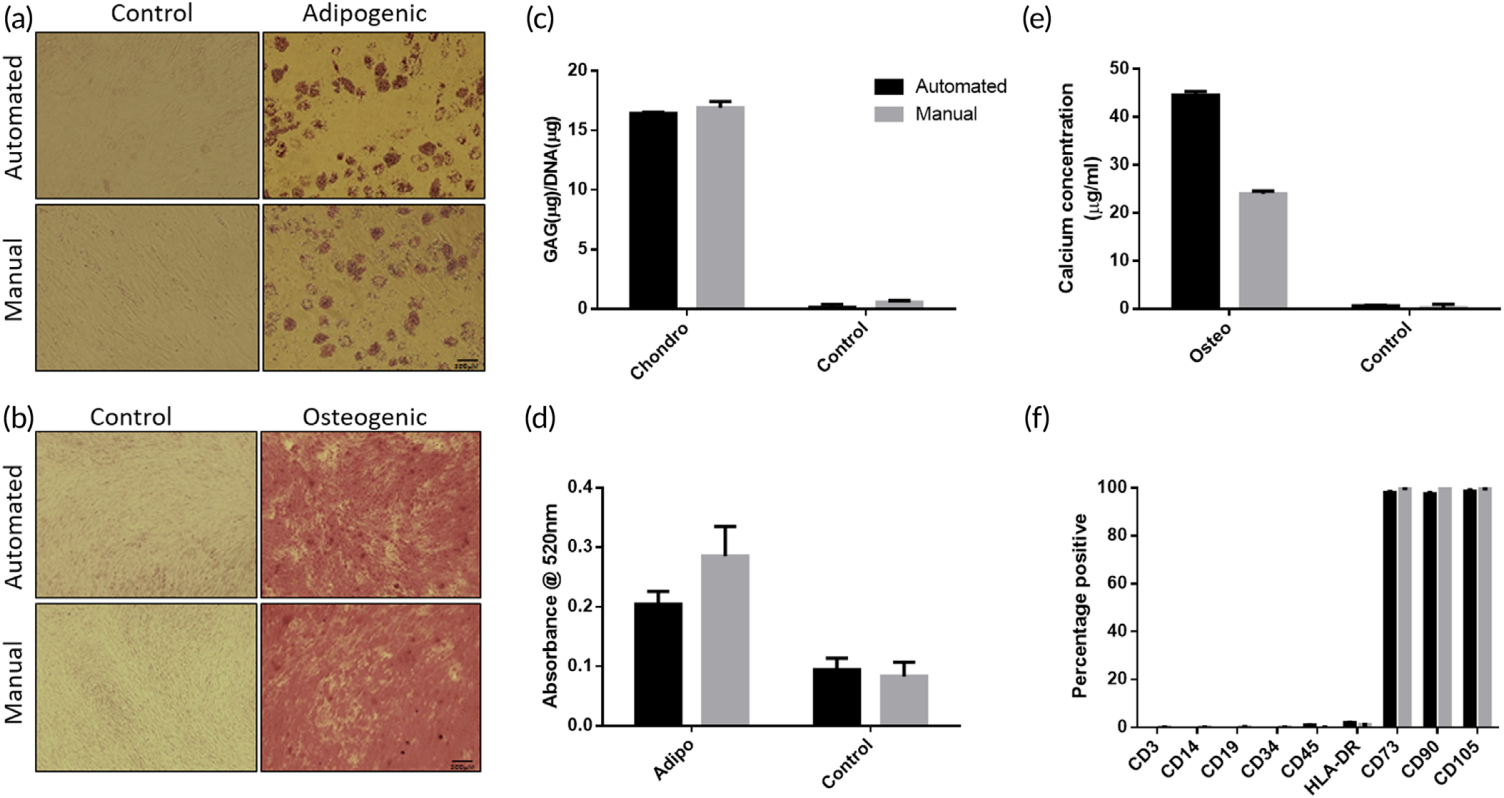
Cell quality assessment postexpansion in the manual and automated bioreactor systems. (a) Oil Red O staining to assess adipogenic differentiation. Scale bars, 200 μm. (b) Alizarin Red staining to assess osteogenic differentiation. Scale bars, 500 μm. (c) GAG/DNA (μg/μg) content for chondrogenic differentiation quantification. (d) Quantification of adipogenic differentiation by extraction of Oil Red O. (e) Quantification of osteogenic differentiation by calcium concentration measurement (μg/ml). (f) Cell surface marker expression assessed by flow cytometry. Data shown as mean ± *SD*, *n* = 3.

Cell quality was assessed to validate the AUTOSTEM platform comparing differentiation and surface immunophenotype to manually produced cells.^23^ MSC chondrogenesis (s‐GAG normalized to DNA levels) was similar in MSCs produced by the platform as well as manually produced cells (Figure 8c). Osteogenic differentiation showed higher calcium deposition by cells produced on the automated platform (Figure 8b,e) with adipogenesis confirmed by Oil Red O positive lipid vacuoles (Figure 8a,d). Cell surface profiles were assessed using ISCT markers^23^ with MSCs generated in the automated and manual systems displaying the expected low expression of hematopoietic lineage markers (<2%) and high expression of CD73, CD90, and CD105 (>98%) (Figure 8f).

### 2.9 hMSC quality assessment postexpansion

A detailed analysis of the materials (reagents and consumable items) required for the manufacture of MSCs using the automated platform as compared to the manual process revealed that consumable costs for the automated system are higher than that for the manual process. Increased costs are associated with the requirement for customized items such as tubing sets specifically designed for the fluidics pathway in the AUTOSTEM platform. However, the labor costs associated with the manual process are greater than those for the automated platform. An estimate of the numbers of personnel hours required to carry out the processes described revealed a reduction of at least 60% for the operation of the automated platform (Table S1). Additionally, facility costs for the automated process are lower than the manual process. The automated platform has been designed to allow it to be located in a grade D background, with the platform itself acting as a self‐contained grade A area. Certain tasks required prior to set up of the automated platform do require the use of a traditional clean room, a grade A hood in a grade B background. These include the preparation of reagents (media, microcarriers), bioreactor assembly, and in the case of the exemplar runs described in this manuscript, the planar culture of cells used to seed the bioreactors. In contrast, the manual process needs carried out with grade A facilities required for all steps associated with the preparation of the bioreactor, cells and media as well as the harvesting and downstream processing. Overall we estimate the use of the automated platform would result in a saving of 35% on the costs association with the manufacture of an MSC batch under GMP conditions. Additionally, it is likely that automated process may result in reduced production run times leading to additional savings in facility and materials costs.

## 3 DISCUSSION

The production of cell‐based ATMPs presents unique challenges not seen in other biologics manufacturing systems. Since the therapeutic product is a living cell, rather than a biomolecule produced by cells, there are sterility limitations as product sterilization is not possible. The production process, from collection of source tissue to final dose filling, must the carried out in an aseptic environment. Vulnerable points where sterility may be breached (tissue collection and operator‐associated process steps) are avoided in the AUTOSTEM platform through the inclusion of a closed controlled marrow collection device and the fully closed robotic configuration leading to a needle‐to‐needle closed process. There is no exposure of the product to contamination risks that are high during manual processing. Inevitable variations in operator practice are also problematic, a consequence of the complex nature of cell processing. Manual processing involving plating, feeding, passage, harvest, filling, and cryopreservation steps requires decisions which often lack objectivity and skill levels vary.

However, robotic‐enabled automation for cell therapies requiring the use of solid tubes or vials will need to be assessed in the context of cryopreservation and ultimate delivery of the therapeutic to patients. Increasing the vial size can be facilitated with validation of cell product quality and stability over time. Another issue is the closed delivery of the cell product to patients. Future iterations of the platform could include the use of specially designed vials of various sizes amenable to robotic handling but with caps incorporating a septum to facilitate closed delivery of the cell product to patients. Design of a metal device with the capacity to hold cryobags and expand in response to increased pressure as cell suspensions are introduced by the robot may also be an option. Both options would enable generation of the appropriate cell suspension by hospital pharmacies for direct injection or infusion by clinicians.

The AUTOSTEM platform fully removes operators from contact with the process. Operator fatigue is also associated with the demanding and repetitive nature of manual cell processing; working in a clean room environment for long hours in sterile gowns/masks is physically and psychologically demanding, and undoubtedly contributes to operator error. Labor commitments associated with 24‐7 processing also adds greatly to production costs.

The essential elements of an automated cell therapy manufacturing platform include:

1. Closed and automated collection of source tissue.
2. Aseptic control achieved through a fully closed process.
3. Highly automated processing using integrated robotics in an operator‐independent design.
4. In‐process checkpoints using real‐time, label‐free protocols to measure process quality and drive decisions regarding critical process steps (e.g., media change, cell passage).
5. 24‐7 operation, adaptable to industry needs.
6. Fully GMP‐ and GAMP‐compliance with integrated software control.
7. Adaptability to bioreactors of varying configuration and scale for different cell types.

AUTOSTEM addresses all these challenges by considering every step in the production cycle, from tissue harvest to process control and final product formulation, and is capable of producing cells that are identical to that generated by traditional methods in terms of yield and phenotype. It avoids the need for expensive clean room infrastructure, minimizes opportunities for introduction of contaminants, reduces operator‐associated risks, facilitates 24‐7 production and significantly reduces production costs.

Additionally, the doors of the system can be opened.

The AUTOSTEM platform focuses on the isolation of adherent hMSCs derived from bone marrow as the exemplar selected for the AUTOSTEM system validation. For this reason, the cell isolation device has been designed specifically for cells from bone marrow. However, the platform itself is readily adaptable for production of adherent hMSCs from sources such as adipose tissue, umbilical cord and other commonly used tissues. Isolation of MSCs from these tissues will require the addition of some minor process steps such as enzymatic digestions/additional wash steps. However, all the additional steps required are readily adaptable to automation and inclusion in the platform. Additionally, T‐ or CAR‐T cells have been shown to have improved growth and functionality in stirred‐tank bioreactor similar to that integrated in the AUTOSTEM platform used in the current study.^24^, ^25^, ^26^ Automation and digitization of production as well as distribution of cell therapies is critical for the future of the industry. As such, AUTOSTEM represents a critical starting point for application of Industry 4.0 Technology^27^ to enable widespread and affordable patient benefit.

## 4 MATERIALS AND METHODS

### 4.1 Planar cell culture

hMSCs were isolated from bone marrow aspirates (Lonza) of healthy donors after informed consent using direct plating^28^ and expanded as previously described.^13^, ^29^, ^30^ An ethical statement was provided by Lonza indicating that permission from all donors for use of their bone marrow in research applications only was obtained under informed consent or legal authorization. The recipient of the marrow sample (Aston University) was approved for isolation of human bone marrow‐derived MSCs by their University Ethics Committee (AHRIC reference number 2017‐PH [HTA]) and the University Research Ethics Committee (UREC, reference 1189). Briefly, P2 cells were seeded at 5000 cells/cm^2^ on tissue culture plastic and cultured in α‐MEM (1 g/L glucose, Glutamax, Gibco, Thermofisher) supplemented with 10% (v/v) foetal bovine serum (Sigma Aldrich) and 1 ng/ml bFGF (Peprotech). Medium changes were performed every 3 days and cells were subcultured every 6 days, when 70%–80% confluency was achieved. Cell harvest was carried out by incubating with Tryple Select (Thermofisher) for 30 min at 37°C and 5% CO_2_ in a humidified incubator. The enzyme was then diluted by the addition of D‐PBS and the cell suspension was centrifuged at 220g for 5 min at room temperature. The obtained cell pellet was then resuspended in a known volume of culture medium and cell counts were performed. Planar cell culture was used to generate the cell inoculation densities for the bioreactor cultures.

### 4.2 Bioreactor cell culture

For the comparison runs, the bioreactor setup was kept as comparable as possible between the manual and the automated experimental configuration. A single use, disposable stirred tank bioreactor vessel (Mobius 3L, Millipore) was used for all experimental runs in conjunction with the EZ‐Control bioreactor control platform (Applikon), probes (pH, temperature, and DO) and other relevant accessories such as the heating mantle and motor adapter (Applikon). The pH probe was calibrated at two points and then autoclaved. Only pH was monitored and control achieved by using a bicarbonate‐based medium and CO_2_ supplementation to aeration when required. The pH was maintained within the range of 7.2–7.6. The DO probe was autoclaved and then calibrated postassembly in the bioreactor. DO was monitored only and not controlled. However, temperature was monitored using a standard temperature probe and also controlled using a heating mantle with the temperature set to 37°C. The single use, disposable bioreactor vessel was then assembled by introducing the presterilized probes, connecting the mixing drive, adaptor and heating mantle, followed by connection of all components to the controller and establishing the gas connection.

The bioreactor was operated at a working volume of 1 and 1.2 L in the manual and automated setup, respectively. On the basis of a detailed earlier assessment of 17 different microcarriers,^31^ xeno‐free Plastic P102L (PALL) were chosen for the bioreactor cultures. These microcarriers were weighed to provide a surface area of 5000 cm^2^ and sterilized by autoclaving, followed by cell inoculation at 6000 cells/cm^2^ (~5 cells/microcarrier).^32^ Aeration was achieved using the headspace air which was found sufficient to satisfy the oxygen demand of hMSCs at densities achievable over a range of bioreactor sizes.^8^, ^32^ Agitation speed was initially set at the minimum speed needed to suspend the microcarriers, *N*_js_, of 50 rpm, a strategy which had proved suitable from spinner flasks^33^ through different sizes and geometries of bioreactors.^34^, ^35^ During the project, it was established that in order to maintain suspension and minimize aggregation as culture progressed, the agitator speed needed to be increased, first to 65 rpm at day 6 and 75 rpm at day 10. This approach has also been adopted successfully when cultivating bovine MSCs.^16^, ^35^ Samples were collected for cell counts, imaging, spent medium analysis and cell quality assessment throughout the process.

#### 4.2.1 Manual versus automated bioprocessing

The sequence diagram for the cell cultivation process highlights minor differences in operation between the manual and automated set‐ups (Figure 4a). Briefly, all manual processing steps were carried out on the bench using a peristaltic pump (Watson‐Marlow 120S), presterilized glass bottles with tubing and a sterile welder (Terumo TSCD‐II) for establishing sterile connections (Figure 4b). The automated process set‐up is shown in Figure 4c. Briefly, the processing steps were carried out using the preassembled tubing sets connected via single‐use sterile connectors (CPC AseptiQuik S Connector) to presterilized glass or polypropylene bottles used for storing medium or other reagents. The inoculum was delivered to the system in the disposable bone‐marrow harvesting tube set (Figure 3b). Inoculum, medium and reagents were transported via positive pressure applied to the reservoir. Liquid transport out of the bioreactor for waste or harvest was facilitated through high‐speed peristaltic pumps (Verder).

#### 4.2.2 Medium exchange

Following protocols similar to those established in earlier manual bioreactor studies,^8^, ^32^ 50% medium exchanges were performed at days 3, 5, and 7 and every day thereafter until the end of the culture.

#### 4.2.3 Cell growth

Cell counts were performed to monitor cell growth throughout the process with microcarrier samples obtained on days 3, 5, 7, 9, and 10 and at harvest. Table S2 compares cell concentrations over time in the manual and automated system (>95% detachment was achieved with viability).

#### 4.2.4 Cell harvest

The cell harvesting step was performed at day 11 in the bioreactor (see Figure 5 for the associated sequence diagram) as described previously.^8^, ^32^, ^34^ Briefly, agitation was stopped and the microcarriers were allowed to settle before removing 50% of the spent medium and replacing with D‐PBS for washes. These steps were repeated three times, followed by removal of 50% of the volume and replacing with Tryple Select 3X (Gibco, ThermoFisher) for 30 min, while stirring at 150 rpm continuously. A sample was then taken for microscopic assessment of cell dissociation from the microcarriers. Once complete cell dissociation was achieved with cells freely floating in suspension, the enzyme was further diluted with D‐PBS to achieve a volume of 1 L.

A sample was taken postdissociation for cell counting and filtration used to separate the cell suspension from the microcarriers, followed by centrifugation to pellet the cells and perform final cell counts. In the automated system the cell suspension was filtered through a single‐use microcarrier separation and filtration device (with pores ≤90 μm) for in‐line retention of microcarriers (Harvestainer™ BioProcess Container, Thermofisher). The microcarrier filtration device was attached to the bioreactor via sterile welding prior to harvesting. The cell‐microcarrier suspension was pumped through the separation device and the filtrate containing only cells transferred to the grade A area (Figure 2c). For manual processing, filtration devices (pores of 100 μm) were used (Steriflip, Millipore) to separate and collect the cells from microcarriers once they have been released by enzymatic treatment.

#### 4.2.5 Analytical methods

Daily imaging was done by phase contrast microscopy. Cell counts and viability were performed using the Nucleocounter NC3000 (Chemometec) as per manufacturer's instructions for two separate samples. Counts were performed directly on the microcarriers using the reagent A100 and reagent B protocol. Briefly, the cell‐microcarrier suspension was diluted to a 1:1:1 ratio with reagent A100 and reagent B (Chemometec); reagent A100 lyses the cells from the microcarriers releasing the nuclei, while reagent B stabilizes the suspension. The resulting suspension was loaded onto a Nucleocassette Via‐1, preloaded with acridine orange and DAPI and the cassette then transferred Nucleocounter NC3000 machine for processing.

Spent medium samples were collected before and after medium exchanges in the bioreactor and were analyzed for glucose and lactate concentrations on an AccuTrend Plus meter (Roche). Fresh growth medium was used as baseline control.

Based on cell counts, the following parameters were calculated:

1. Specific growth rate
(1) μ=lnCxtCx0∆t,
  where *μ* is the specific growth rate (h^−1^), *Cx*(*t*) and *Cx*(0) represent cell numbers at the end and start of the culture, *t* represents time in culture (h).
2. Doubling time
(2) $$\mathrm{td}=\frac{\ln2}{\mu },$$
  where *t*_*d*_ is doubling time (h) and *μ* is the specific growth rate (h^−1^).
3. Fold increase
(3) $$\mathrm{FI}=\frac{\mathrm{Cx}\left(t\right)}{\mathrm{Cx}\left(0\right)},$$
  where *Cx*(*t*) represents the maximum cell number and *Cx*(0) is the initial cell number.

### 4.3 Cell characterization

Cell quality postexpansion was assessed by multilineage differentiation and flow cytometry for cell surface marker expression. The multilineage differentiation capacity of the hMSC cultures was assessed by their differentiation to the classical MSC lineages, that is, chondrogenic, osteogenic, and adipogenic using established protocols.^36^ All reagents were supplied by Sigma Aldrich unless stated otherwise. Chondrogenic potential was examined using a 3D pellet culture system. Briefly, 2.5 × 10^5^ cells were placed in a screw‐capped 1.5 ml tube, washed in incomplete chondrogenic medium (ICM) consisting of DMEM (Sigma Aldrich) containing 4.5 g/L glucose, 2 mM glutamine, 100 mM dexamethasone, 50 μg/ml ascorbic acid, 40 μg/ml l‐Proline, 1% ITS+ (Insulin, Transferrin, Selenium) (Corning), 1 mM sodium pyruvate. The cells were pelleted by centrifugation at 100g for 5 min and then cultured in complete chondrogenic medium (CCM), that is, ICM supplemented with 10 ng/ml TGF‐β3 (Peprotech). For quantification, at day 21, pellets were digested with papain overnight. The sulfated glycosaminoglycan (s‐GAG) content of the digested pellets was assessed by DMMB (1,9‐dimethylmethylene blue) (Sigma Aldrich) binding at pH 1.5. DNA content was measured using Pico Green (Invitrogen). Results were presented as GAG levels per pellet and calculated as a ratio of the amount of DNA per pellet. For histological analysis, pellets were fixed in 10% neutral buffered formalin, submitted to histological processing using a Leica ASP300S automatic tissue processor and embedded in paraffin. Cut sections (5 μm) were stained with Safranin O and Fast Green FCF. Slides were mounted in DPX mounting solution (Sigma) and imaged with an Olympus BX43 microscope.

For osteogenesis, cells were seeded in hMSC culture medium and transferred at 90% confluence to osteogenic medium comprising DMEM (1 g/L glucose; Sigma Aldrich), 2 mM l‐glutamine, 100 nM dexamethasone, 100 μM ascorbic acid, 10 mM β‐glycerophosphate, 10% FBS (Hyclone) and penicillin–streptomycin (100 U/ml). Medium replaced every 3–4 days for up to 14 days, when monolayers were fixed with 10% ice cold methanol, then stained with 2% Alizarin Red and imaged using an Olympus IX71 microscope. For quantitative measurement of calcium, the cell monolayers were scraped into 0.5 M hydrochloric acid. The calcium levels were determined using the Calcium CPC Liquicolor kit (Stanbio Inc.).

hMSC adipogenic potential was assessed in confluent cultures incubated in adipogenic induction medium comprising DMEM (4.5 g/L glucose; Sigma Aldrich), 2 mM l‐glutamine, 10% FBS (Hyclone), 1 μM dexamethasone, 10 μg/ml insulin (Roche), 200 μM indomethacin, 500 μM 3‐isolbutyl‐1‐methylxanthine and penicillin–streptomycin (100 U/ml). After 3 days, the culture was transferred to adipogenic maintenance medium comprising DMEM (4.5 g/L glucose), 10% FBS (Hyclone), 10 μg/ml insulin (Roche) and penicillin–streptomycin (100 U/ml) for 1 day. This cycle was repeated three times after which the cells were maintained in adipogenic maintenance medium for a further 5 days. Cell monolayers were fixed in 10% neutral buffer formalin and stained with 0.18% Oil Red O in 60% isopropanol before imaging using an inverted Olympus IX71 microscope. The Oil red O stain was extracted from the stained cell monolayers using isopropanol and measured by absorbance at 520 nm.

Surface marker expression of hMSCs was carried out by flow cytometry using the BD FACS Canto II flow cytometer (BD Biosciences) using antibodies against CD3, CD14, CD19, CD34, CD45, HLA‐DR and the MSC positive markers CD73, CD90 and CD105 (BD Biosciences) as described previously.^28^ Postacquisition analysis was carried out using the FlowJo software (Treestar Inc.).

### 4.4 Data analysis

All analyses were performed in duplicate. Cell counts for every time point were acquired from two independent samples from each replicate. For cell differentiation assays, cells obtained from the manual and automated processes were seeded into multiple wells and triplicate wells were quantitatively analyzed. Cell surface analysis by flow cytometry was also carried out on both the manual and automated cell products. For each cell product, three independently stained samples for each antibody were analyzed. Results are shown as the mean with errors calculated as one standard deviation.

## 5 CONCLUSIONS

Although ATMPs, whether cell or gene therapies represent the future for patient care for numerous intractable disease scenarios, a significant impediment to widespread availability worldwide is manufacturing capacity and cost of goods. The necessity to produce functional therapeutic cells at clinically relevant numbers at a competitive cost is not possible using manual production systems. The AUTOSTEM platform represents a full GMP‐enabled production suite for the automated and closed production of adherent cells. The platform is distinct in this area of stem cell manufacturing, enabling full end‐to‐end automation from procurement of source tissue to cryopreservation of the final cell product. Although developed for isolation and expansion of bone marrow‐derived MSCs, AUTOSTEM is easily adaptable to a wide array of bioreactor configurations for both adherent and nonadherent cells and represents a significant advance in automated therapeutic cell processing to address patient needs.

## AUTHOR CONTRIBUTIONS

**Jelena Ochs:** Conceptualization (equal); data curation (supporting); formal analysis (supporting); funding acquisition (supporting); investigation (equal); methodology (supporting); project administration (supporting); resources (supporting); software (supporting); supervision (equal); validation (supporting); visualization (supporting); writing – original draft (equal); writing – review and editing (supporting). **Mariana P. Hanga:** Conceptualization (supporting); data curation (supporting); formal analysis (supporting); investigation (supporting); methodology (supporting); project administration (supporting); resources (supporting); supervision (supporting); validation (supporting); visualization (supporting); writing – original draft (supporting); writing – review and editing (equal). **Georgina Shaw:** Conceptualization (supporting); data curation (equal); formal analysis (equal); funding acquisition (supporting); investigation (supporting); methodology (supporting); project administration (equal); resources (supporting); software (supporting); supervision (supporting); validation (supporting); visualization (supporting); writing – original draft (supporting); writing – review and editing (supporting). **Niamh Duffy:** Conceptualization (supporting); formal analysis (supporting); investigation (supporting); methodology (supporting); resources (supporting). **Michael Kulik:** Conceptualization (supporting); data curation (supporting); formal analysis (supporting); funding acquisition (supporting); methodology (supporting); supervision (supporting). **Nokilaj Tissin:** Data curation (supporting); formal analysis (supporting); investigation (supporting); methodology (supporting). **Daniel Reibert:** Formal analysis (supporting); investigation (supporting); methodology (supporting). **Ferdinand Biermann:** Data curation (supporting); formal analysis (supporting); investigation (supporting); software (supporting); validation (supporting); visualization (supporting). **Panagiota Moutsatsou:** Data curation (supporting); formal analysis (supporting); investigation (supporting); methodology (supporting); validation (supporting). **Shibani Ratnayake:** Data curation (supporting); investigation (supporting); methodology (supporting). **Alvin Nienow:** Conceptualization (supporting); data curation (supporting); formal analysis (supporting); investigation (supporting); methodology (supporting); supervision (supporting); validation (supporting); visualization (supporting); writing – original draft (supporting); writing – review and editing (supporting). **Niels Koenig:** Conceptualization (equal); funding acquisition (equal); investigation (supporting); methodology (supporting); project administration (supporting); resources (supporting); supervision (equal); visualization (supporting); writing – original draft (supporting); writing – review and editing (supporting). **Robert Schmitt:** Conceptualization (supporting); funding acquisition (supporting); investigation (supporting). **Qasim Rafiq:** Data curation (equal); formal analysis (equal); investigation (equal); methodology (equal); supervision (equal); writing – original draft (equal); writing – review and editing (equal). **Frank Barry:** Conceptualization (equal); data curation (supporting); formal analysis (supporting); funding acquisition (equal); investigation (supporting); methodology (supporting); supervision (equal); writing – original draft (equal); writing – review and editing (equal). **Christopher J. Hewitt:** Data curation (supporting); formal analysis (supporting); funding acquisition (supporting); investigation (supporting); methodology (supporting); supervision (equal). **J. Mary Murphy:** Conceptualization (lead); data curation (supporting); formal analysis (equal); funding acquisition (lead); investigation (equal); methodology (equal); project administration (equal); resources (lead); software (supporting); supervision (equal); validation (equal); visualization (equal); writing – original draft (equal); writing – review and editing (lead).

### PEER REVIEW

The peer review history for this article is available at https://publons.com/publon/10.1002/btm2.10387.

## Supporting information


**Figure S1** Photographs of the physical AUTOSTEM system. (1) Bioreactors with control units; (2) pump system including squeeze valves and peristaltic pumps; (3) cooled storage area with medium heater coils; (4) grade A area with robot, pipettor and disposable storage units; (5) uncapping of a centrifugation bottle; (6) close‐up of the automated pipettor filling 5 ml cryovials; (7) loading of the cell counter cassette; (8) material transfer hatch; and (9) robot loading a cryo‐container into the −80°C freezer.

**Figure S2** Screenshots of the graphical user interface of the control software of the AUTOSTEM system. (a) Main Tab of the software showing an overview of all devices and their status. (b) Tab showing details for bioreactor station, including status and individual services offered by the device.

**Figure S3** Screenshots of the recipe builder of the control software of the AUTOSTEM system. Recipe builder of the control level software in which protocols can be built from sequences of individual services.

**Video S1** Video with impressions of the AUTOSTEM system in action. The video shows clips of the automated process, including operation of the bioreactor, sampling from the bioreactor, preparation and filling of cryovials and storage of the vials in the −80°C freezer. See https://www.youtube.com/watch?v=GMQTK8JpcQ8.

**Table S1** Comparison of production costs and staff time required for manual versus automated processing.

**Table S2** Cell concentrations over time in culture in the manual and automated processes. Data shown as the average of two separate runs (d—days in culture).

## Data Availability

Data sharing not applicable to this article as no datasets were generated or analysed during the current study.

## References

[1] Rafiq QA , Hewitt CJ . Cell therapies: why scale matters. Pharm Bioprocess. 2015;3(2):97‐99.

[2] Harrison RP , Medcalf N , Rafiq QA . Cell therapy‐processing economics: small‐scale microfactories as a stepping stone toward large‐scale macrofactories. Regen Med. 2018;13(2):159‐173. 29509065 10.2217/rme-2017-0103

[3] Coelho A , Damasio Alvites R , Vieira Branquinho M , Guerreiro SG , Mauricio AC . Mesenchymal stem cells (MSCs) as a potential therapeutic strategy in COVID‐19 patients: literature research. Front Cell Dev Biol. 2020;8:602647. 33330498 10.3389/fcell.2020.602647 PMC7710935

[4] Barry F . MSC therapy for osteoarthritis: an unfinished story. J Orthopaedic Res. 2019;37:1229‐1235. 10.1002/jor.24343 31081558

[5] Thomas RJ , Chandra A , Liu Y , Hourd PC , Conway PP , Williams DJ . Manufacture of a human mesenchymal stem cell population using an automated cell culture platform. Cytotechnology. 2007;55:31‐39. 19002992 10.1007/s10616-007-9091-2 PMC2289788

[6] Williams DJ , Thomas RJ , Hourd PC , et al. Precision manufacturing for clinical‐quality regenerative medicines. Philos Transact A Math Phys Eng Sci. 2012;370:3924‐3949. 10.1098/rsta.2011.0049 22802496

[7] Thomas RJ , Chandra A , Hourd PC , Williams DJ . Cell culture automation and quality engineering: a necessary partnership to develop optimized manufacturing processes for cell‐based therapies. JALA: J Assoc Lab Autom. 2008;13(3):152‐158. doi:10.1016/j.jala.2007.12.003

[8] Nienow AW , Coopman K , Heathman TRJ , Rafiq QA , Hewitt CJ . Bioreactor engineering fundamentals for stem cell manufacturing. In: Cabral JMS et al., eds. Stem Cell Manufacturing. Elsevier Science, Chapter 3; 2016:43‐76 ISBN 978‐0‐444‐63265‐4.

[9] Moutsatsou P , Ochs J , Schmitt RH , Hewitt CJ , Hanga MP . Automation in cell and gene therapy manufacturing: from past to future. Biotechnol Lett. 2019;41:1245‐1253. 31541330 10.1007/s10529-019-02732-z PMC6811377

[10] Rafiq QA , Twomey K , Kulik M , et al. Developing an automated robotic factory for novel stem cell therapy production. Regener Med. 2016;11(4):351‐354. 10.2217/rme-2016-0040 27168080

[11] Xiao K , Hou F , Huang X , Li B , Qian ZR , Xie L . Mesenchymal stem cells: current clinical progress in ARDS and COVID‐19. Stem Cell Res Therapy. 2020;11:305. 10.1186/s13287-020-01804-6 PMC7373844 32698898

[12] Callens S , Twomey K , Rafiq QA , et al. Developing an automated robotic factory for novel stem cell therapy production. Regen Med. 2016;11(4):351‐354. 27168080 10.2217/rme-2016-0040

[13] Rafiq QA , Hanga MP , Heathman TRJ , et al. Process development of multipotent stromal cell microcarrier culture using an automated high‐throughput microbioreactor. Biotechnol Bioeng. 2017;114(10):2253‐2266. 28627713 10.1002/bit.26359 PMC5615370

[14] Harrison RP , Ruck S , Rafiq QA , Medcalf N . Decentralised manufacturing of cell and gene therapy products: learning from other healthcare sectors. Biotechnol Adv. 2018;36(2):345‐357. 29278756 10.1016/j.biotechadv.2017.12.013

[15] Jung S , Ochs J , Kulik M , König N , Schmitt RH . Highly modular and generic control software for adaptive cell processing on automated production platforms. Proc CIRP. 2018;72:1245‐1250.

[16] Murphy M , Barry F , Leschke C , et al. The AUTOSTEM platform for closed manufacture of bone marrow‐derived mesenchymal stromal cells using a closed, scalable and automated robotic system. Cytotherapy. 2017;19(5):S122.

[17] Ochs J , Barry F , Schmitt R , Murphy JM . Advances in automation for the production of clinical‐grade mesenchymal stromal cells: the AUTOSTEM robotic platform. Cell Gene Therapy Insights. 2017;3(8):739‐748.

[18] Eagle H , Levine EM . Growth regulatory effects of cellular interaction. Nature. 1967;213:1102‐1106. 6029791 10.1038/2131102a0

[19] Wilson A , Webster A , Genever P . Nomenclature and heterogeneity: consequences for the use of mesenchymal stem cells in regenerative medicine. Regen Med. 2019;14(6):595‐611. 31115266 10.2217/rme-2018-0145 PMC7132560

[20] Schop D , Janssen FW , van Rijn LDS , et al. Growth, metabolism and growth inhibitors of mesenchymal stem cells. Tissue Eng Part A. 2009;15(8):1877‐1886. 19196147 10.1089/ten.tea.2008.0345

[21] Qie S , Liang D , Yin C , et al. Glutamine depletion and glucose depletion trigger growth inhibition via distinctive gene expression reprogramming. Cell Cycle. 2012;11(19):3679‐3690. 22935705 10.4161/cc.21944 PMC3478318

[22] Dominici M , Le Blanc K , Mueller I , et al. Minimal criteria for defining multipotent mesenchymal stromal cells. The International Society for Cellular Therapy position statement. Cytotherapy. 2006;8(4):315‐317. 16923606 10.1080/14653240600855905

[23] Klarer A , Smith D , Cassidy R , Heathman TRJ , Rafiq QA . Demonstrating scalable T‐cell expansion in stirred‐tank bioreactors. Bioprocess Int. 2018;16(6):6‐14.

[24] Costariol E , Rotondi M , Amini A , et al. Establishing the scalable manufacture of primary human T‐cells in an automated stirred‐tank bioreactor. Biotechnol Bioeng. 2019;116(10):2488‐2502. 31184370 10.1002/bit.27088

[25] Costariol E , Rotondi M , Amini A , et al. Demonstrating the manufacture of human CAR‐T cells in an automated stirred‐tank bioreactor. Biotechnol J. 2020;15(9):2000177‐1‐2000177‐13. 10.1002/biot.202000177 32592336

[26] Alcacer V , Cruz‐Machado V . Scanning the industry 4.0: a literature review on technologies for manufacturing systems. Eng Sci Technol Int J. 2019;22(3):899‐919.

[27] Ferro F , Spelat R , Shaw G , et al. Survival/adaptation of bone marrow‐derived mesenchymal stem cells after long‐term starvation through selective processes. Stem Cells. 2019;37(6):813‐827. 30835892 10.1002/stem.2998

[28] Heathman TRJ , Rafiq QA , Chan AKC , et al. Characterisation of human mesenchymal stem cells from multiple donors and the implications for large scale bioprocess development. Biochem Eng J. 2016;108:14‐23.

[29] Fábián Z , Ramadurai S , Shaw G , et al. Basic fibroblast growth factor modifies the hypoxic response of human bone marrow stromal cells by ERK‐mediated enhancement of HIF‐1α activity. Stem Cell Res. 2014;12(3):646‐658. 24667757 10.1016/j.scr.2014.02.007

[30] Rafiq QA , Coopman K , Nienow AW , Hewitt CJ . Systematic microcarrier screening and agitated culture conditions improve human mesenchymal stem cell yield in bioreactors. Biotechnol J. 2016;11:473‐486. 26632496 10.1002/biot.201400862 PMC4991290

[31] Rafiq QA , Brosnan KM , Coopman K , Nienow AW , Hewitt CJ . Culture of human mesenchymal stem cells on microcarriers in a 5 L stirred‐tank bioreactor. Biotechnol Lett. 2013;35(8):1233‐1245. 23609232 10.1007/s10529-013-1211-9

[32] Hewitt CJ , Lee K , Thomas RJ , Smith M , Nienow AW , Thomas CR . Expansion of human mesenchymal stem cells on microcarriers. Biotechnol Lett. 2011;33:2325‐2335. 21769648 10.1007/s10529-011-0695-4

[33] Nienow AW , Hewitt CJ , Heathman TRJ , et al. Agitation conditions for the culture and detachment of hMSCs from microcarriers in multiple bioreactor platforms. Biochem Eng J. 2016;108:24‐29.

[34] Hanga MP , Ali J , Moutsatsou P , et al. Bioprocess development for scalable production of cultivated meat. Biotechnol Bioeng. 2020;117:3029‐3039. 32568406 10.1002/bit.27469

[35] Murphy JM , Dixon K , Beck S , Fabian D , Feldman A , Barry F . Reduced chondrogenic and adipogenic activity of mesenchymal stem cells from patients with advanced osteoarthritis. Arthritis Rheum. 2002;46(3):704‐713. doi:10.1002/art.10118 11920406

[36] Hanga MP , de la Raga FA , Moutsatsou P , Hewitt CJ , Nienow A , Wall I . Scale‐up of an intensified bioprocess for the expansion of bovine adipose‐derived stem cells (bASCs) in stirred tank bioreactors. Biotechnol Bioeng. 2021;118(8):3175‐3186. 34076888 10.1002/bit.27842

