# Supplementary figures and images for: Needle to needle robot‐assisted manufacture of cell therapy products
Source: Bioeng Transl Med. 2022 Aug 6;7(3):e10387. doi: 10.1002/btm2.10387 (PMC9472012; doi:10.1002/btm2.10387)

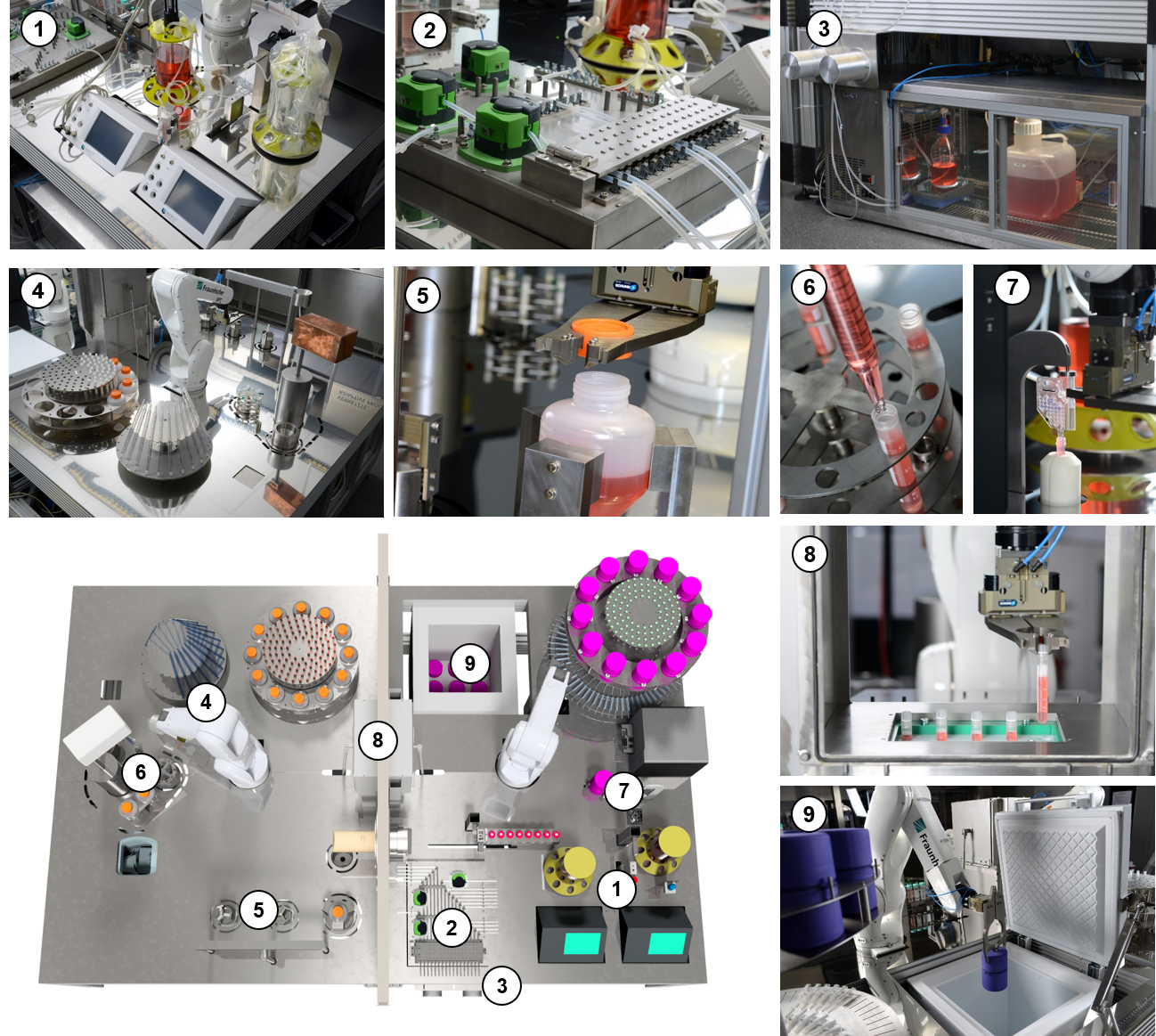

Supplement: Supplementary file 1 — Figure S1 Photographs of the physical AUTOSTEM system. (1) Bioreactors with control units; (2) pump system including squeeze valves and peristaltic pumps; (3) cooled storage area with medium heater coils; (4) grade A area with robot, pipettor and disposable storage units; (5) uncapping of a centrifugation bottle; (6) close‐up of the automated pipettor filling 5 ml cryovials; (7) loading of the cell counter cassette; (8) material transfer hatch; and (9) robot loading a cryo‐container into the −80°C freezer. [file BTM2-7-e10387-s004.png]

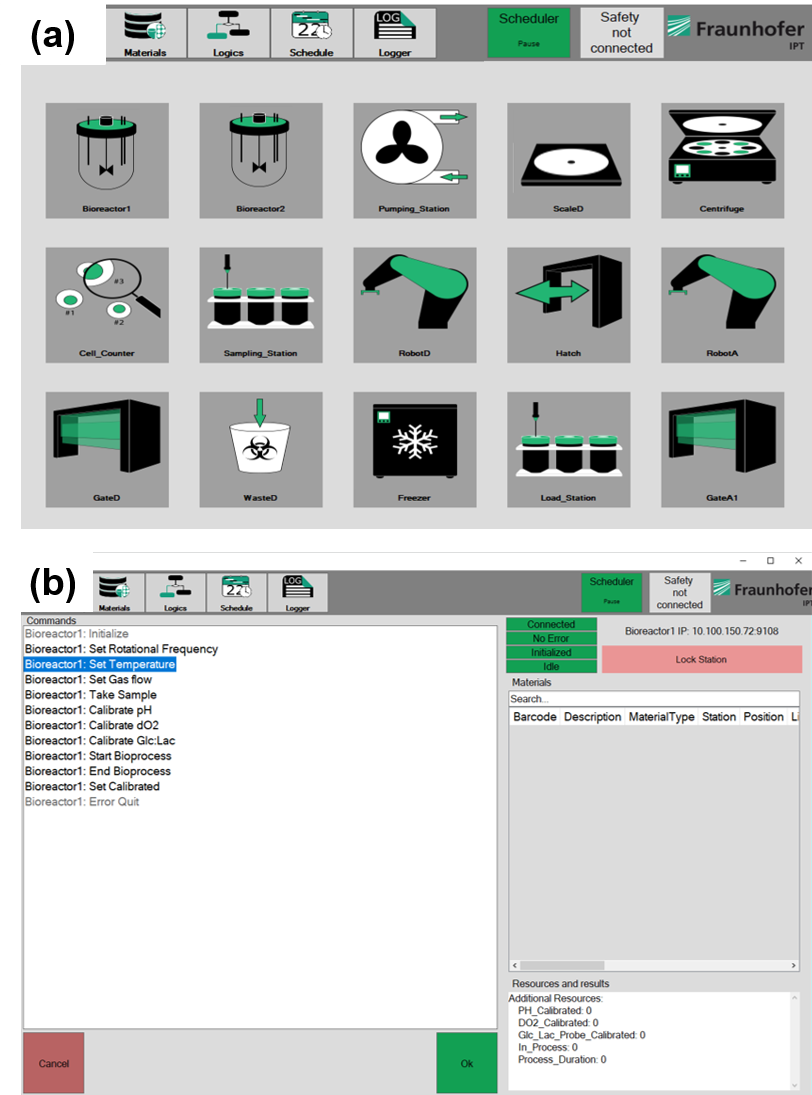

Supplement: Supplementary file 2 — Figure S2 Screenshots of the graphical user interface of the control software of the AUTOSTEM system. (a) Main Tab of the software showing an overview of all devices and their status. (b) Tab showing details for bioreactor station, including status and individual services offered by the device. [file BTM2-7-e10387-s006.png]

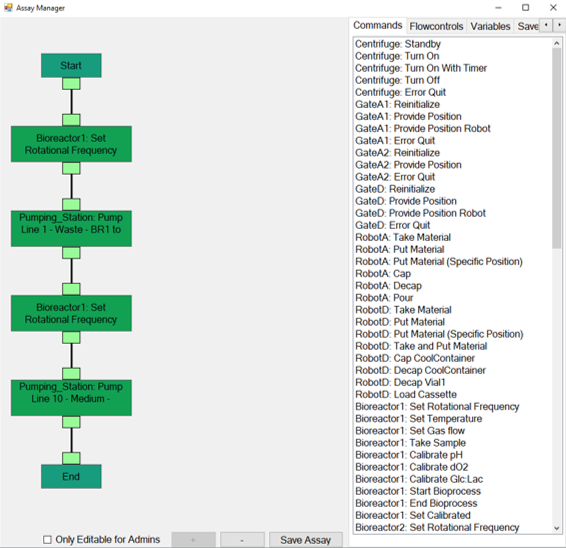

Supplement: Supplementary file 3 — Figure S3 Screenshots of the recipe builder of the control software of the AUTOSTEM system. Recipe builder of the control level software in which protocols can be built from sequences of individual services. [file BTM2-7-e10387-s002.png]
