# Supplementary material for: Needle to needle robot‐assisted manufacture of cell therapy products
Source: Bioeng Transl Med. 2022 Aug 6;7(3):e10387. doi: 10.1002/btm2.10387 (PMC9472012; doi:10.1002/btm2.10387)
Supplement: Supplementary file 5 — Table S1 Comparison of production costs and staff time required for manual versus automated processing. [file BTM2-7-e10387-s005.docx]

Table S1 Comparison of production costs and staff time required for manual versus automated processing.

| **Production Costs (€)** | | |
| --- | --- | --- |
|  | **Manual** | **Automated** |
| Cost of materials | 8,000 | 9,500 |
| Cost of facility | 36,000 | 25,000 |
| Cost of staffing | 8,500 | 3,500 |
| **Total costs** | **52,500** | **34,000** |
| **Staff Time (Hours)** | | |
|  | **Manual** | **Automated** |
| Planar culture process | 14 | 14 |
| Preparation of media/ micro-carriers/ bioreactor set-up | 4 | 4 |
| Platform preparation | - | 4 |
| Platform running | - | 11 |
| Seeding manual process | 4 | - |
| Sampling of bioreactors | 20 | - |
| Feeding of bioreactors | 32 | - |
| Harvesting and freezing | 10 | - |
| **Total required staff time** | **84** | **33** |
