# Supplementary material for: Needle to needle robot‐assisted manufacture of cell therapy products
Source: Bioeng Transl Med. 2022 Aug 6;7(3):e10387. doi: 10.1002/btm2.10387 (PMC9472012; doi:10.1002/btm2.10387)
Supplement: Supplementary file 6 — Table S2 Cell concentrations over time in culture in the manual and automated processes. Data shown as the average of two separate runs (d—days in culture). [file BTM2-7-e10387-s001.docx]

| Manual | |  | Automated | |  |
| --- | --- | --- | --- | --- | --- |
| Time | cells/mL | Stdev | Time | cells/mL | Stdev |
| [Days] | [10^5^ cells/mL] | [10^5^ cells/mL] | [Days] | [10^5^ cells/mL] | [10^5^ cells/mL] |
| 3 | 0.31 | 0.10 | 3 | 0.97 | 0.01965 |
| 5 | 0.91 | 0.35 | 5 | 2.91 | 0.162 |
| 7 | 1.54 | 0.62 | 7 | 4.21 | 1.5915 |
| 8 | 3.08 | 0.77 | 8 | 3.94 | 0.6315 |
| 9 | 3.79 | 1.13 | 9 | 3.98 | 0.267 |
| 10 | 3.85 | 0.52 | 10 | 3.11 | 0.567 |
| 11 | 5.83 | 0.56 | 11 | 4.82 | 0.7785 |
| After dissociation | 6.24 | 0.23 | After dissociation | 7.12 | 1.26 |

**Table S2**. Cell concentrations over time in culture in the manual and automated processes. Data shown as the average of 2 separate runs.
